# Growing vascular endothelial cells express somatostatin subtype 2 receptors

**DOI:** 10.1054/bjoc.2001.1881

**Published:** 2001-07

**Authors:** J C Watson, D A Balster, B M Gebhardt, T M O'Dorisio, M S O'Dorisio, G D Espenan, G J Drouant, E A Woltering

**Affiliations:** Departments of Surgery, Ophthalmology, Radiology, The Stanley S. Scott Cancer Center, The Neuroscience Center of Excellence, Louisiana State University Health Sciences Center, New Orleans, Louisiana, 70112; Departments of Medicine and, Pediatrics, The Ohio State University, Columbus, Ohio, 43210; The Veterans Administration Medical Center, New Orleans, Louisiana, 70112, USA

**Keywords:** somatostatin, somatostatin receptors, receptor subtypes, angiogenesis, vascular endothelium, gene expression

## Abstract

We hypothesized that non-proliferating (quiescent) human vascular endothelial cells would not express somatostatin receptor subtype 2 (sst 2) and that this receptor would be expressed when the endothelial cells begin to grow. To test this hypothesis, placental veins were harvested from 6 human placentas and 2 mm vein disks were cultured in 0.3% fibrin gels. Morphometric analysis confirmed that 50–75% of cultured vein disks developed radial capillary growth within 15 days. Sst 2 gene expression was determined by reverse transcription-polymerase chain reaction (RT-PCR) analysis of the RNA from veins before culture and from tissue-matched vein disks that exhibited an angiogenic response. The sst 2 gene was expressed in the proliferating angiogenic sprouts of human vascular endothelium. The presence of sst 2 receptors on proliferating angiogenic vessels was confirmed by immunohistochemical staining and in vivo scintigraphy. These results suggest that sst 2 may be a unique target for antiangiogenic therapy with sst 2 preferring somatostatin analogues conjugated to radioisotopes or cytotoxic agents. © 2001 Cancer Research Campaign http://www.bjcancer.com


British Journal of Cancer (2001) **85**(2), 266–272
© 2001 Cancer Research Campaign
doi: 10.1054/ bjoc.2001.1881, available online at http://www.idealibrary.com on IDEAL®

http://www.bjcancer.com

### Growing vascular endothelial cells express somatostatin subtype 2 receptors

**JC Watson[1], DA Balster[7], BM Gebhardt[2,5], TM O'Dorisio[6], MS O'Dorisio[7], GD Espenan[3], GJ Drouant[1] and EA Woltering[1,4,5,8]**

Departments of Surgery[1], Ophthalmology[2] and Radiology[3], The Stanley S. Scott Cancer Center[4], The Neuroscience Center of Excellence[5], Louisiana State University Health Sciences Center, New Orleans, Louisiana 70112; The Ohio State University Departments of Medicine[6] and Pediatrics[7], Columbus, Ohio 43210, and The Veterans Administration Medical Center[8], New Orleans, Louisiana 70112, USA

**Summary** We hypothesized that non-proliferating (quiescent) human vascular endothelial cells would not express somatostatin receptor subtype 2 (sst 2) and that this receptor would be expressed when the endothelial cells begin to grow. To test this hypothesis, placental veins were harvested from 6 human placentas and 2 mm vein disks were cultured in 0.3% fibrin gels. Morphometric analysis confirmed that 50–75% of cultured vein disks developed radial capillary growth within 15 days. Sst 2 gene expression was determined by reverse transcription-polymerase chain reaction (RT-PCR) analysis of the RNA from veins before culture and from tissue-matched vein disks that exhibited an angiogenic response. The sst 2 gene was expressed in the proliferating angiogenic sprouts of human vascular endothelium. The presence of sst 2 receptors on proliferating angiogenic vessels was confirmed by immunohistochemical staining and in vivo scintigraphy. These results suggest that sst 2 may be a unique target for antiangiogenic therapy with sst 2 preferring somatostatin analogues conjugated to radioisotopes or cytotoxic agents. © 2001 Cancer Research Campaign http://www.bjcancer.com

**Keywords**: somatostatin; somatostatin receptors; receptor subtypes; angiogenesis; vascular endothelium; gene expression

Angiogenesis is a tightly regulated process that normally occurs during embryonic development and is suppressed in adult life. We have previously demonstrated that somatostatin (SRIF) analogues will inhibit angiogenesis in the chicken chorioallantoic membrane (CAM) and in the rabbit corneal micropocket models (Woltering et al, 1991; Patel et al, 1994; Barrie et al, 1993; Conway et al, 1996). These observations have been confirmed by Danesi et al, who demonstrated that somatostatin analogues will inhibit angiogenesis in the CAM model and will inhibit the proliferation of human umbilical vein endothelial cells (HUVECs) in culture (Danesi et al, 1997). In our previous studies, we demonstrated that SRIF-induced inhibition of angiogenesis in the CAM is G-protein-adenylate cylase-, and calcium-dependent (Patel et al, 1994). We have also shown that the ability of a somatostatin analogue to inhibit angiogenesis is proportional to its sst 2-binding affinity and its ability to inhibit growth hormone release, a sst 2-dependent process (Woltering et al, 1997). These observations imply that endothelial cells growing from human blood vessels express sst 2. External scintigraphic scanning with the sst 2-preferring analogue, $^{111}$In-pentetreotide, has demonstrated binding to cells in malignant tissue and to cells in non-malignant tissue in which angiogenesis is occurring but not to normal blood vessels. Similarly, van Hagen et al demonstrated that $^{111}$In-pentetreotide localizes to active rheumatoid joints but not to osteoarthritic joints (van Hagen et al, 1994). These authors postulated that the radiolabelled somatostatin analogue binds to the vascular pannus in active rheumatoid joints but does not bind to cells in non-vascularized, normal or osteoarthritic joints (van Hagen et al, 1994). Reubi et al, using autoradiographic techniques have demonstrated that proliferating human peritumoral vessels bind $^{125}$I-tyr$^3$-octreotide, a sst 2-preferring radioligand, while distant quiescent vessels do not (Reubi et al, 1994, 1996; Denzler and Reubi, 1999).

Based on these observations, we hypothesized that non-proliferating human vascular endothelial cells do not express sst 2 but these receptors are expressed when endothelial cells proliferate. To test this hypothesis, normal placental veins were harvested from 6 human placentas obtained immediately following delivery. RNA was harvested from quiescent (native) placental vessels and from vessel fragments which had been cultured in vitro. In this culture system, we have investigated changes in gene expression as vascular endothelial cells begin to sprout, migrate, and proliferate (Watson et al, 1996). Previous work in our laboratories using this culture system revealed that the gene for a vascular endothelial growth factor (VEGF) receptor, *kdr*, is expressed in angiogenic vein disks but not in placental vessels in which endothelial growth is not occurring.

*Received 23 October 2000*
*Revised 13 March 2001*
*Accepted 15 March 2001*

*Correspondence to:* EA Woltering

### METHODS

### In vitro culture of human placental veins

Fresh human placentas were obtained from 6 anonymous donors following guidelines established by the LSU Institutional Review Board, Protocol #2828. Placental veins were trimmed of surrounding tissue, opened longitudinally and 2 mm diameter disks prepared using a sterile skin biopsy punch. Residual placental vein tissue was placed in Ultraspec® RNA isolation solution (Biotex, Inc, Houston, TX) for subsequent RNA extraction. Vein disks (N = 100/assay) were incorporated into 0.3% fibrin gels in complete tissue culture medium containing 20% fetal bovine serum (FBS), ε-amino caproic acid, antibiotics and antifungals. Fibrin gels were overlaid with tissue culture medium consisting of minimal essential medium (MEM, Gibco, Grand Island, NY), supplemented with 10% FBS, penicillin, streptomycin and Fungizone according to methods outlined by Watson et al and Brown et al (Brown et al, 1996; Watson et al, 1996). Culture plates were incubated in a 5% $CO_2$/95% air atmosphere at 37°C for 15 days and observed daily with an inverted microscope. At 15 days, cultures exhibiting endothelial cell growth and those not exhibiting an angiogenic response were collected for RNA isolation. Separate experiments with cultured, nonangiogenic vein disks were performed to determine if their failure to proliferate was due to poor cell viability. The MTT cell viability assay (Promega Corporation, Madison, WI) confirmed that these disks contained viable cells even though they did not exhibit an angiogenic response.

### Reverse transcription-polymerase chain reaction (RT-PCR) analysis of gene expression

Venous tissue collected at the time of preparation of the disks, cultured disks that did not develop endothelial sprouts and disks which developed sprouts were collected in separate RNase-free tubes and homogenized in an RNA isolation solution (RNAzol, Biotex, Houston, TX). RNA from each sample was quantitated by $A_{260}/A_{280}$ spectrophotometric analysis. 500 ng of RNA was reverse transcribed in the presence of random hexamer primers using a reverse transcription system (Perkin-Elmer Applied Biosystems, Carson City, CA). The expression of the sst 2 gene and the constitutively expressed gene for glyceraldehyde-6-phosphodehydrogenase (GAPDH) was determined by amplifying the cDNAs from non-proliferating and from proliferating placental endothelial cells. Following 35 amplification cycles, the gene products were analysed on 2% agarose gels stained with ethidium bromide. Visualization of the amplified gene products was performed with an Eagle-EyeII® gel documentation system (Stratagene, Inc, La Jolla, CA) (Figure 1). Nucleotide sequences of the primers and probes used are given in Table 1.

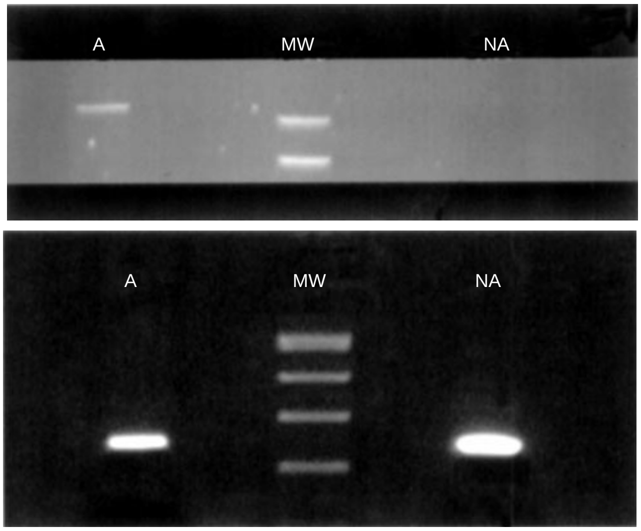

**Figure 1** (**A**) Reverse transcription-polymerase chain reaction analysis of non-angiogenic (NA) and an angiogenic (A) vein disk for sst 2 transcripts. MW, molecular weight markers. (**B**) Reverse transcription-polymerase chain reaction analysis of non-angiogenic (NA) and an angiogenic (A) vein disk for GAPDH transcripts. MW, molecular weight markers. The source of the mRNAs for this reaction was the same as used in A

**Table 1** Primers and probes used in this study

| | |
|---|---|
| **Primers** | |
| sst 2 antisense | CAG TTC AGA TAC TGG TTT GGA G |
| sst 2 sense | AGC CAT GGA CAT GGC GGA TGA G |
| GAPDH antisense | GTC ATG AGC CCT TTC ACG ATG C |
| GAPDH sense | GAA TCT ACT GGC GTC TTC AC C |
| **Probes** | |
| sst 2 | GAG GTC AAA TGG AAT GGA TAG CCA TGT GTG GCT TCC ATT GAG |
| GAPDH | GTG ATG GGT GTC AAC CAC GAC |

*Primers and probes were synthesized by the Core Laboratories of the LSU Health Sciences Center.

### Southern hybridization

Southern hybridization was used to confirm the identity of the amplified gene products. DNA probes, complementary to an internal sequence of the amplified sst 2 and GAPDH amplicons (Table 1), were labelled with biotin (Ready-To-Go beads, Pharmacia Biotech, Piscataway, NJ). 10 mg of labelled probe in Express Hyb® hybridization solution was incubated with nitrocellulose membranes (Tropilon Plus, Tropix Inc, Bedford, MA) to which were bound the amplified products. The hybridized biotin-labelled probes were detected using the Southern-Light® detection system (Tropix). Autoradiography was performed on X-ray film (X-OMAT AR, Kodak Corp., Rochester, NY).

### Localization of radiolabelled somatostatin analogues in angiogenic vessels in vivo

Nude mice (Harlan Sprague Dawley, Indianapolis, IN) were injected in the hindquarter with $1 \times 10^6$ SKNSH human neuroblastoma tumour cells, in accordance with LSUHSC Institutional Animal Care and Use Committee approved Protocol #1806. SKNSH cells do not express sst 2 as measured by both RT-PCR and in vitro binding assays (O'Dorisio et al, 1994). Injection sites were observed for 2–3 months until tumours grew to 2 cm in diameter. Mice were injected in the tail vein with 3 mCi of $^{125}$I-WOC4a[DTyr-Tyr-Tyr-DTyr-Cys-Phe-DTrp-Lys-Thr-Cys-Thr], an sst 2-preferring somatostatin analogue (Woltering et al, 1998). We have previously shown that this radioiodinated analogue has a high binding affinity for sst 2 (Kd~1nM) and will induce receptor-specific cytotoxicity in human (IMR32) neuroblastoma cells (Meyers et al, 1998). Background counts were allowed to clear for 5 days, and the mice were then scanned and radiographed. Because of the low energy emissions of $^{125}$I, mice were scanned on both their ventral and dorsal surfaces.

### Preparation of sst 2 antiserum

An oligonucleotide corresponding to the N terminal 45 amino acids of sst 2 was cloned into pET-32a(+) vector (Norvagen, Madison, WI) as a C terminal fusion to thioredoxin using T4DNA ligase (TA cloning kit, Invitrogen, Carlsbad, CA). The resulting plasmid was sequenced in order to confirm that the insert was in the correct orientation and that the sequence matched the native gene sequence. The plasmid was transformed into the AD494 bacterial host strain (Novagen, Madison, WI) and plated on LB plates containing ampicillin and kanamycin overnight at 37°C.

© 2001 Cancer Research Campaign 

Colonies were picked and cultured in 20 ml LB medium (containing 100 μg ml$^{-1}$ ampicillin and 30 μg ml$^{-1}$ kanamycin) overnight at 37°C in a shaking incubator. Bacterial cultures were diluted 1:50 with fresh medium and cultured 3 to 4 hours until the O.D. at 600 nm was greater than 0.6. Isopropylthio-β-D-galactosidase (IPTG) was added to a final concentration of 1 mM to induce protein expression. After 2 hours bacteria were collected by centrifugation at 8000 ***g*** for 10 minutes. The pellets were resuspended in start buffer (1 × PBS and 10 Mm imidazole, pH 7.4) and sonicated using a Polytron® at setting 7 with 5 second bursts for 1 minute to release the sst 2 peptide. Supernatants were collected and pellets sonicated again. The sst 2-containing supernatant was filtered through a 0.45 μm filter (Nalgene, Rochester, NY) and the protein concentration was determined. The peptide was purified using the Pharmacia His Trap® column system (Amersham Pharmacia Biotech, Piscataway, NJ), dialysed against a 0.9% NaCl solution overnight at 4°C, and concentrated using a Centriplus® concentrator (Amicon, Beverly, MA).

3 kg male rabbits were immunized with 500 μg sst 2 peptide in 0.5 ml 0.9% NaCl emulsified with an equal volume of Freund's complete adjuvant. The emulsion was injected both intramuscularly and intradermally. Four booster injections (100 μg peptide) were given at 3-week intervals. Serum was collected before each booster injection and 3 weeks after the final injection and stored at –20°C. This antibody has been previously characterized by Balster et al (2001) and Albers et al (2000).

### Immunohistochemical staining

Placental vein disks were cultured in 48-well plate membrane-bottomed inserts. Disks which visually exhibited an angiogenic response were fixed in 10% neutral-buffered formalin and embedded in paraffin using standard tissue processing techniques. Serial 4 μm slices were obtained and every tenth slide stained with haematoxylin and eosin until the portion of the block containing the vessel disk was reached. Factor VIII staining was performed to confirm that vascular endothelial cells were present in the outgrowths.

Paraffin sections of the vessel/fibrin clot were deparaffinized and incubated in 3% $H_2O_2$ for 15 minutes at room temperature, washed in distilled water ($dH_2O$) and placed and preheated in Antigen Retrieval® (Biogenix, San Ramon, CA) solution. Slides were microwaved for 10 minutes on medium power and left in sealed container for 15 minutes, followed by washing in $dH_2O$ 3 times and then in OptiMax Wash Buffer® (Biogenix). Slides were incubated in PeroxiDaze 1® (Biocare Medical, Walnut Creek, CA) for 15 minutes at room temperature, again washed in OptiMax buffer, and incubated in Power Block® (Biogenix, San Ramon, CA) for 10 minutes. A 1:1000 dilution of sst 2 antiserum (or a 1:1000 dilution of preimmune serum) in diluent buffer (Biomedia, Foster City, OH) was added to separate slides and incubated at 4°C overnight. Slides were washed in OptiMax Wash Buffer® for 20 minutes at room temperature. Secondary antibody (Biogenix multilink super sensitive biotinylated antibody) was added to the sections which were incubated for 20 minutes at room temperature. Slides were rinsed in OptiMax Wash Buffer® for a minimum of 20 minutes, followed by incubation in conjugated streptavidin (Biogenix) for 20 minutes. Slides were rinsed in OptiMax Wash Buffer® for 20 minutes. AEC (3-amino-9-ethyl-carbazole) chromogen was applied for 10 to 20 minutes, followed by rinsing in OptiMax Wash Buffer® and $dH_2O$. Slides were lightly counterstained with haematoxylin for 10 seconds followed by 3 seconds in ammonia water, rinsed in $dH_2O$, coverslipped with Crystal mount, placed in a 60°C oven for 15 minutes and the cover slip sealed with Permount (Biogenix, San Ramon, CA).

### RESULTS

### Gene expression in quiescent, proliferating and non-proliferating placental vein cultures

We analysed sst 2 receptor mRNA expression in non-proliferating vein and proliferating placental vein endothelial cells obtained from 6 placentas. In each of the placental samples, we found that sst 2 gene expression was absent in non-proliferating endothelial cells but was present in the proliferating endothelium (Figure 1A). GAPDH mRNA was expressed in all samples (Figure 1B). Sst 2 gene expression was confirmed by Southern hybridization analysis of the amplified gene products derived from proliferating placental vein endothelial cells. Hybridization of the amplified gene product with a sst 2 probe revealed a single product of the expected molecular weight (Figure 2).

### Localization of radiolabelled somatostatin analogues in angiogenic vessels in vivo

Scans and radiographs were performed in register so that we could compare relative radioligand uptake in the liver (normal route of excretion) (Figure 3A) and in the tumour (Figure 3B). These scans revealed significant radioligand uptake in the SKNSH tumour even though the tumour cells do not express somatostatin receptors. Alternatively, normal (non-proliferating) blood vessels in non-tumour-bearing sites did not accumulate the radioligand, implying that binding of the radioligand in the tumour was due to sst 2 receptor expression on the angiogenic blood vessels supplying the tumour.

### Immunohistochemical staining of vein disks

To confirm the endothelial nature of the sprouts from the vein disks, factor VIII, staining was performed (Figure 4). Immunohistochemical staining for sst 2 receptors revealed the presence of the receptor protein on cells forming the vascular sprouts (Figure 5). The vein disk did not stain for sst 2 receptors except at the periphery of the disk, the origin of the neovascular sprouts. This staining pattern was not seen in the non-angiogenic disks incubated in the anti-sst 2 antiserum (not shown). Neither the angiogenic nor the non-angiogenic vein disks exhibited staining following incubation in the nonimmune serum (Figure 6). Previous work with this

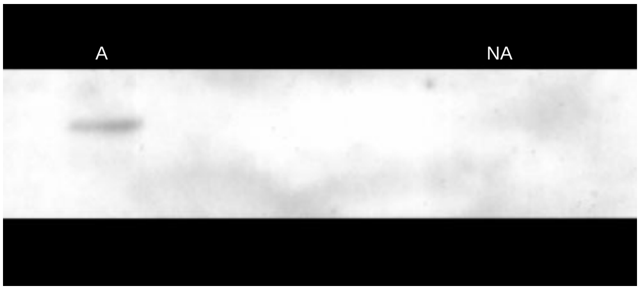

**Figure 2** Southern hybridization of the RT-PCR product shown in Figure 1 with an sst 2 probe demonstrates a single product of the appropriate molecular weight in the mRNA from the angiogenic (A), but not in the mRNA from the non-angiogenic (NA) vein disk

© 2001 Cancer Research Campaign

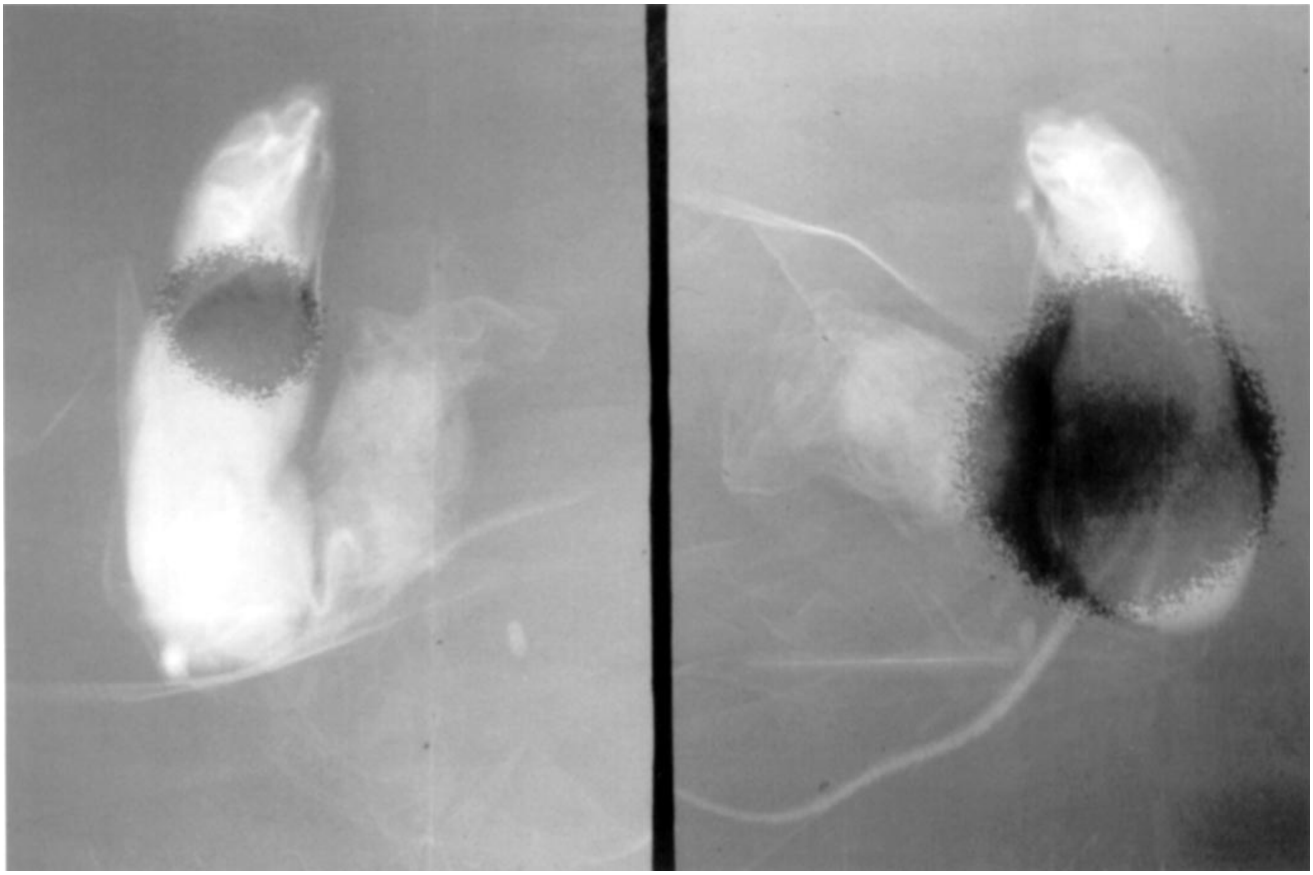

**Figure 3** (**A**) Scintographs and radiographs were performed in register to compare relative radioligand uptake in the liver (normal route of excretion). The SKNSH tumour (sst 2-negative) shows intense uptake of a radiolabelled sst 2-preferring somatostatin analogue. (**B**) Scintographs and radiographs were performed in register to compare relative radioligand uptake in the tumour. The SKNSH tumour (sst 2-negative) shows intense uptake of a radiolabelled sst 2-preferring somatostatin analogue antibody demonstrated sst 2 staining on the endothelial cells of peritumoral vessels in a mouse tumour xenograft model. Absorption of the sst 2 antibody with sst 2 antigen ablated the immunohistochemical staining of the peritumoral vessels (Albers et al, 2000).

### DISCUSSION

We have previously demonstrated in both the chicken chorioallantoic membrane (CAM) assays and the rabbit bFGF-induced corneal micropocket angiogenesis models that octreotide acetate will inhibit angiogenesis (Woltering et al, 1991; Barrie et al, 1993; Patel et al, 1994; Conway et al, 1996). This inhibition is proportional to a somatostatin analogue's ability to inhibit growth hormone which in turn is proportional to the analogue's binding affinity for sst 2 (Woltering et al, 1997). Inhibition of angiogenesis in the CAM model is G-protein-, adenylate cyclase-, and calcium-dependent similar to somatostatin inhibition of peptide release from neuroendocrine cells (Patel et al, 1994). Based on these observations, it appears that similar receptors and post-receptor signal transduction pathways are responsible for somatostatin's ability to inhibit peptide release, tumour growth, and angiogenesis (Bhatena et al, 1981; Thompson et al, 1986; Woltering et al, 1986; Wynick and Bloom, 1991).

The widespread use of radiolabelled somatostatin analogues in patients with neuroendocrine tumours has demonstrated that these sst 2-containing tumours avidly bind radiolabelled sst 2-preferring somatostatin analogues such as tyr$^{3}$-octreotide, lanreotide, and pentetreotide (Schirmer et al, 1993; Woltering et al, 1994; Martinez et al, 1995; Woltering et al, 1995; McCarthy et al, 1998; Cuntz et al, 1999; Espenan et al, 1999). Inevitably, as larger numbers of tumour-bearing patients have been scanned with these radiolabelled analogues, tissues and organs subjected to non-tumour disease processes have been shown to bind these radioligands. In a study by van Hagen et al, $^{111}$In-pentetreotide localized to joints actively involved in rheumatoid disease (van Hagen et al, 1994). This is in contrast to a lack of binding seen in osteoarthritic joints. One of the prominent features of rheumatoid arthritis is the development of a vascular pannus in the active rheumatoid joint, a feature absent in normal joints, quiescent rheumatoid joints, and osteoarthritis joints. Vascularization of the rheumatoid joint is felt to contribute to joint destruction seen in rheumatoid arthritis. Localization of pentetreotide to the active rheumatoid, vascularized joint is consistent with our observation that neovessel formation is associated with expression of sst 2.

Several authors have demonstrated that radiolabelled sst 2-preferring somatostatin analogues will bind to a variety of tumour cell types, including breast cancer, endocrine tumours, renal cell carcinomas, melanomas, neuroblastomas, medulloblastomas, high-grade gliomas, high-grade lymphomas, meningiomas, Merkel cell tumours, and small cell carcinomas of the lung (Woltering et al, 1995). More recently, others have demonstrated

© 2001 Cancer Research Campaign

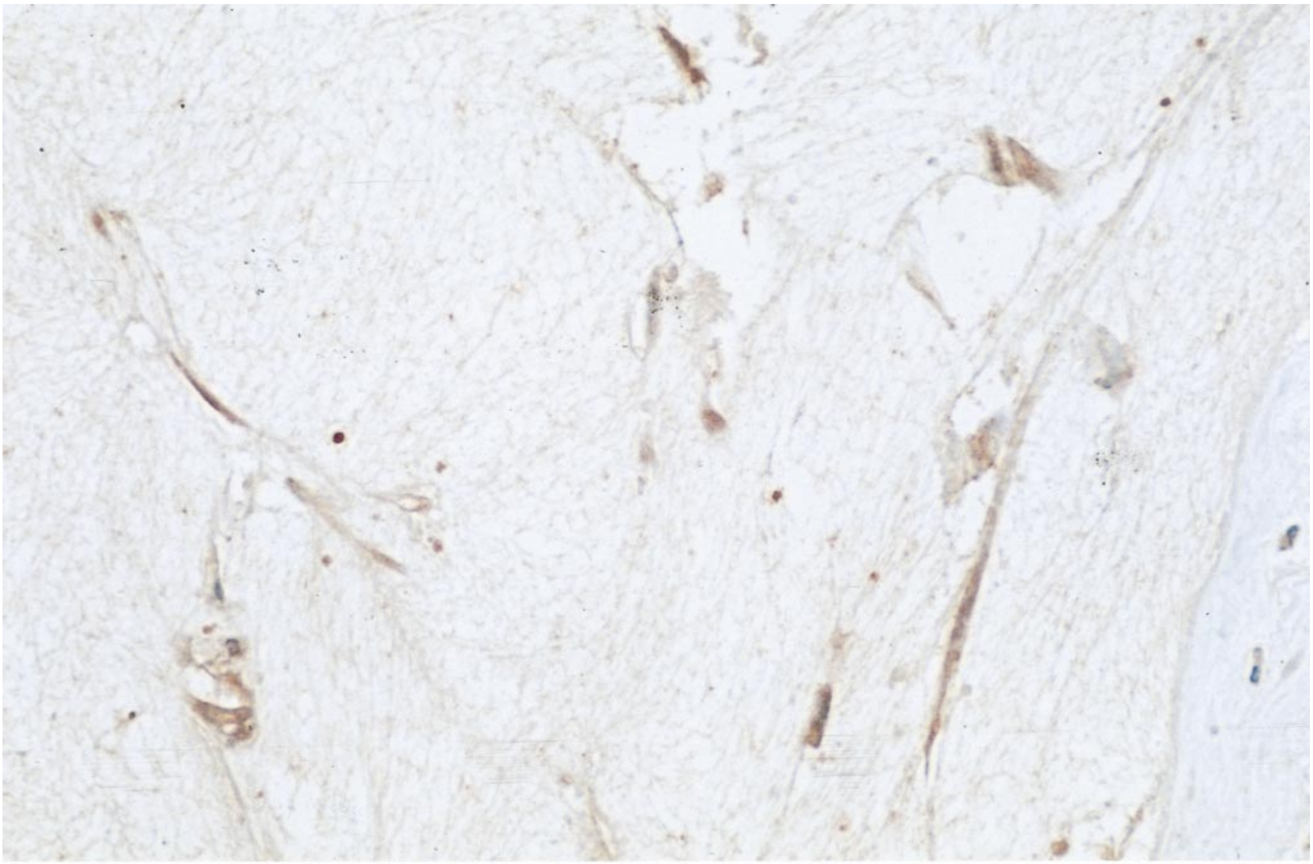

**Figure 4** Factor VIII staining of endothelial cell sprouts from a vein disk (40X)

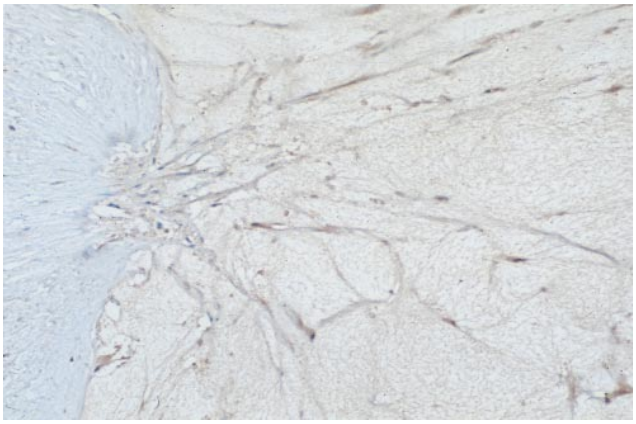

**Figure 5** Immunohistochemical stain for sst 2 receptors on endothelial cell sprouts (20X)

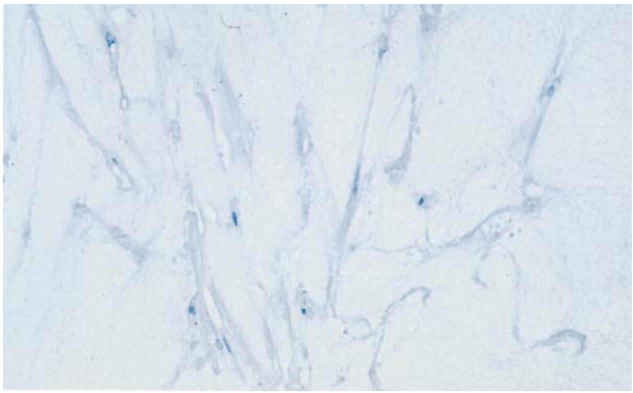

**Figure 6** Absence of staining following incubation of a section of an angiogenic disk in nonimmune serum (40X)

that prostate cancers will selectively bind radiolabelled somatostatin analogues, however, the level of binding is less than that seen in endocrine neoplasms (Nilsson et al, 1995; Kalkner et al, 1998). Other tumours have been imaged with radiolabelled analogues and in many instances subsequent evaluation of these tumours by autoradiography shows little or no uptake or binding of the radiolabelled analogue by tumour cells. We believe that radiolabelled analogue binding to tumour-associated angiogenic blood vessels may explain tumour imaging in sst 2-negative tumours. In almost all tumour types, high-grade lesions have more intense ligand binding than low-grade lesions, an observation consistent with a poorly differentiated tumour developing a more intense angiogenic response.

These findings may have considerable clinical relevance. An angiogenic response is required for a tumour to grow beyond a diameter of 2 mm. The expression of sst 2 in angiogenic vessels implies that tumours greater than 2 mm in diameter might bind sst 2-preferring radiolabelled somatostatin analogues either to their

*© 2001 Cancer Research Campaign*

tumour cells, their angiogenic neovessels, or both. This observation provides encouragement for the development of specific sst 2 targeted therapy in tumours with homogeneous or heterogeneous receptor distribution.

Other investigators have noted that tumour cell expression of sst 2 is heterogeneous and that radiolabelled somatostatin analogue therapy is best accomplished by the use of high-energy radioisotopes (Bootsma et al, 1993). While the heterogeneous expression of sst 2 on tumour cells is ubiquitous, it appears that all proliferative neovessels homogeneously express this receptor. This difference in receptor expression may permit the use of low energy radioisotopes for therapy (McCarthy et al, 1998; Espenan et al, 1999). Sst 2-targeted applications may include intraoperative gamma localization of occult primary tumours, and intraoperative gamma detection of microscopically positive primary tumour resection margins (Schirmer et al, 1993; Woltering et al, 1994; Martinez et al, 1995; Cuntz et al, 1999). This sensitive technique may also allow the detection of small deposits of tumour in lymph nodes or in areas outside the normal field of resection (Woltering et al, 1994). Other applications of sst 2-targeted techniques include external scintigraphic imaging and in situ radiotherapy. Recent reports on the use of low energy Auger electron emitters, such as $^{111}$In pentetreotide for therapy of sst 2-expressing tumours are encouraging (McCarthy et al, 1998; Espenan et al, 1999). Others have used high-energy radiolabelled somatostatin analogues for therapy, hoping that higher energy radioisotopes will induce cell death in adjacent receptor-negative cells (Zamora et al, 1997; Espenan et al, 1999). Somatostatin analogues can also be conjugated to chemotherapeutic agents or toxins and may provide highly targeted tumoricidal or angiocidal therapy (Plownoski et al, 2000).

The results of this study reveal that sst 2 expression occurs when vascular endothelial cells begin to grow. The finding paves the way for the development of a highly specific target for anti-angiogenic therapy using sst 2-preferring somatostatin analogues.

### ACKNOWLEDGEMENTS

Supported in part by NIH/NEI core grant #P30 EY02377 to the Department of Ophthalmology, Louisiana State University Health Sciences Center

### REFERENCES

Albers AR, O'Dorisio MS, Balster DA, Caprara M, Gosh P, Chen F, Hoeger C, Rivier J, Wenger GD, O'Dorisio TM and Qualman SJ (2000) Somatostatin receptor gene expression in neuroblastoma. *Reg Peptides* **88**: 61–73

Balster DA, O'Dorisio MS, Summers MA and Turman MA (2001) Segmental expression of somatostatin receptor subtypes sst 1 and sst 2 in tubules and glomeruli of human kidney. *Am J Physiol Renal Physiol* **280(3)**: F457–F465

Barrie R, Woltering EA, Hajarizadeh H, Mueller C, Ure T, Hill N and Fletcher WS (1993) Inhibition of angiogenesis by somatostatin and somatostatin-like compounds is structurally dependent. *J Surg Res* **55**: 446–453

Bhatena SJ, Louis J, Schechter GP, Redman RS, Wahl L and Recant L (1981) Identification of human leukocytes bearing receptors for somatostatin and glucagon. *Diabetes* **30**: 127–131

Bootsma A, van Eijck CH, Schouten KK, Reubi JC, Waser B, Foekens JA, van Pel R, Zwarthoff E, Lamberts SW and de Klein A (1993) Somatostatin receptor-positive primary breast tumors: genetic, patient and tumor characteristics *Int J Cancer* **54**: 357–361

Brown KJ, Maynes SF, Bezos A, Maguire DJ, Ford MD and Parish CR (1996) Novel *in vitro* assay for human angiogenesis. *Lab Invest* **75**: 539–541

Conway MD, Sonmez M, Yank D, Woltering EA and Peyman GA (1996) Inhibition of corneal angiogenesis by octreotide acetate (OA) in a rabbit micropocket model. *Invest Ophthalmol Vis Sci* **37**: 5547–5548

Cuntz MC, Levine EA, O'Dorisio TM, Watson JC, Wray DA, Espenan GD, McKnight CA, Meier JR, Weber LJ, Mera R, O'Dorisio MS and Woltering EA (1999) Intraoperative gamma detection of $^{125}$I-lanreotide in women with primary breast cancer. *Ann Surg Oncol* **6**: 367–370

Danesi R, Agen C, Benelli U, Paolo AD, Nardini D, Bocci O, Campagni A and Tacca MD (1997) Inhibition of experimental angiogenesis by the somatostatin analogue octreotide acetate (SMS 201–995). *Clin Cancer Res* **3**: 263–267

Denzler B and Reubi JC (1999) Expression of somatostatin receptors in peritumoral veins of human tumors. *Cancer* **85(1)**: 188–198

Espenan GD, Nelson JA, Fisher DR, Diaco DS, McCarthy KE, Anthony LB, Maloney TJ and Woltering EA (1999) Experiences with high-dose radiopeptide therapy: the health physics perspective. *Health Physics* **76**: 225–231

Kalkner KM, Nilsson S and Westlin JE (1998) [$^{111}$In-DTPA-D-Phel]-octreotide scintigraphy in patients with hormone-refractory prostatic adenocarcinoma can predict therapy outcome with octreotide treatment: a pilot study. *Anticancer Research* **18(1B)**: 513–516

Martinez DA, O'Dorisio MS and O'Dorisio TM (1995) Intraoperative detection and resection of occult neuroblastoma: a technique exploiting somatostatin receptor expression. *J Pediat Surg* **30**: 1580–1585

McCarthy KE, Woltering EA, Espenan GD, Cronin M, Maloney TJ and Anthony CT (1998) In situ radiotherapy with $^{111}$In-pentetreotide: initial observations and future directions. *The CJSci American* **4**: 94–99

Meyers MO, Anthony CT, Coy DH, Murphy WA, Drouant GJ, Fuselier J, Espenan GD, Maloney TJ and Woltering EA (1998) Multiply radioiodinated somatostatin analogs induce receptor-specific cytotoxicity. *J Surg Res* **76**: 154–157

Nilsson S, Reubi JC, Kalkner KM, Laissue JA, Horisberger U, Olerud C and Westlin JE (1995) Metastatic hormone-refractory prostatic adenocarcinoma expresses somatostatin receptors and is visualized *in vivo* by [$^{111}$In]-labeled DTPA-D-[Phe1]-octreotide scintigraphy. *Cancer Res* **55(**23 Suppl**)**: 5805s–5810s

O'Dorisio MS, Chen F, Wray DA, Qualman SJ and O'Dorisio TM (1994) Identification of somatostatin receptors on human neuroblastoma tumors. *Cell Growth and Differentiation* **5**: 1–8

Patel PC, Barrie R, Hill N, Landeck S, Kurozawa D and Woltering EA (1994) Post-receptor signal transduction mechanisms involved in octreotide-induced inhibition of angiogenesis. *Surgery* **116**: 1148–1154

Plonowski A, Schally AV, Nagy A, Kiaris H, Hebert F and Halmos G (2000) Inhibition of metastatic renal cell carcinomas expressing somatostatin receptors by a targeted cytotoxic analogue of somatostatin AN-238. *Cancer Res* **60**(11): 2996–3001

Reubi JC, Horisberger U and Laissue JA (1994) High density of somatostatin receptors in veins surrounding human cancer tissue: role in tumor-host interactions? *Int J Cancer* **56**: 681–689

Reubi JC, Schaer JC, Laissue JA and Waser B (1996) Somatostatin receptors and their subtypes in human tumors and peritumoral vessels. *Metabolism: Clinical and Experimental* **8**(Suppl 1): 39–49

Schirmer WJ, O'Dorisio TM, Schirmer TP, Mojzisik CM, Hinkle GH and Martin EW (1993) Intraoperative localization of neuroendocrine tumors with $^{125}$I-TYR(3)-octreotide and a hand-held gamma-detecting probe. *Surg* **114**: 745–749

Thompson CS, O'Dorisio TM and Woltering EA (1986) Calcium reverses octreotide inhibition of insulin and glucagon levels in patients with insulinoma and glucagonoma. *Digestion* **27**(Suppl 1): 62–65

van Hagen PM, Markusse HM, Lamberts SWJ, Kwekkenboom DJ, Reubi JC and Krenning EP (1994) Somatostatin receptor imaging: the presence of somatostatin receptors in rheumatoid arthritis. *Arthritis Rheum* **37**: 1521–1528

Watson JC, Gebhardt BM, Alperin-Lea RC, Redmann JC and Woltering EA (1996) Initiation of *kdr* gene transcription is associated with the conversion of human vascular endothelium to an angiogenic phenotype. *Surg Forum* **47**: 462–466

Woltering EA, Ellison EC, O'Dorisio TM, Sparks J, Howe B and Dyben T (1986) Somatostatin-like peptides alter calcium but not secretin sensitivity of gastrinoma cells. *J Surg Res* **40**: 605–608

Woltering EA, Barrie R, O'Dorisio TM, Arce D, Ure C, Cramer A, Holmes D, Roberston J and Fassler J (1991) Somatostatin analogs inhibit angiogenesis in the chick chorioallantoic membrane. *J Surg Res* **50**: 245–251

Woltering EA, Barrie R, O'Dorisio TM, O'Dorisio MS, Nance R and Cook DM (1994) Detection of occult gastrinomas with iodine 125-labeled lanreotide and intraoperative gamma detection. *Surg* **116(6)**: 1139–1141

Woltering EA, O'Dorisio MS and O'Dorisio TM (1995) The role of somatostatin analogs in the management of cancer patients. In *Principles and Practice of Oncology Updates* DeVita VT Jr. (ed) pp. 31–47. Lippincott Healthcare Publications: Philadelphia

© 2001 Cancer Research Campaign 

Woltering EA, Alperin-Lea RC, Sharma C, Keenan EJ, Kurozawa D and Barrie R (1997) Somatostatin analogs: angiogenesis inhibitors with novel mechanisms of action. *Invest New Drugs* **15**: 77–88

Woltering EA, O'Dorisio MS, Murphy WA, Chen F, Drouant GJ, Espenan GD, Fisher DR, Sharma C, Diaco DS, Maloney TJ, Fuselier J, Nelson JA, O'Dorisio TM and Coy DH (1999) Synthesis and characterization of multiply-tyrosinated, multiply-iodinated somatostatin analogs. *J Peptide Res* **53**: 201–206

Wynick D and Bloom SR (1991) The use of long-acting somatostatin analog octreotide in the treatment of gut neuroendocrine tumors. *J Endocrinol Metabol* **73**: 1–4

Zamora PO, Marek MJ and Knapp FF Jr (1997) Preparation of 188Re-RC-160 somatostatin analog: a peptide for local/regional radiotherapy. *Appl Rad & Isotopes* **48(3)**: 305–309

© 2001 Cancer Research Campaign
